# A Randomized Double-Blind Clinical Trial Evaluates the Efficacy of Alternative Herbal Mouthwashes

**DOI:** 10.7759/cureus.40394

**Published:** 2023-06-13

**Authors:** Anjali Oak, Dhanjibhai Bachubhai Sapariya, Chandni Nayak, AV Sunil Kumar Reddy, Regula Sri Lakshmi, Dhanashree Dalal

**Affiliations:** 1 Department of Conservative Dentistry and Endodontics, College of Dental Sciences and Research Centre, Manipur, IND; 2 Ayurvedic Medicine, Government Ayurvedic Hospital, Amreli, IND; 3 Department of Conservative Dentistry and Endodontics, World Dental Specialities, Mumbai, IND; 4 Department of Conservative Dentistry and Endodontics, All India Institute of Medical Sciences, Raipur, IND; 5 Department of Conservative Dentistry and Endodontics, MNR Dental College and Hospital, Hyderabad, IND; 6 Department of Paedodontics and Preventive Dentistry, Dr. D. Y. Patil Dental College and Hospital, Pune, IND

**Keywords:** neem, salvadora persica, chlorhexidine, green tea, dental plaque, herbal mouthwash

## Abstract

Introduction: The removal of dental plaque is the primary step in achieving good oral hygiene. Mechanical plaque removal measures have questionable efficacy in inaccessible areas and proximal aspects of the oral cavity. Out of the several types of mouthwash available, chlorhexidine (CHX) is regarded as the gold standard, but considering its adverse effects, herbal alternatives are being sought after. So, the aim of the study was to evaluate the efficacy of alternative herbal mouthwashes.

Materials and methods: In this randomized, double-blind study, 125 selected patients were divided into five groups. Group 1: negative control - distilled water (DW); group 2: positive control - hexidine mouthwash (ICPA, Gujarat, India); group 3: FeelFresh Herbal Mouthcare Gargle (Able Exports, Gujarat, India); group 4: HiOra mouthwash (Himalaya Wellness Company, Bengaluru, India); group 5: Colgate MaxFresh Fresh Tea mouthwash (Colgate-Palmolive, India). Plaque index (PI), gingival index (GI), and microbial count (CFU) were evaluated before and after the intervention.

Results: The difference among the three types of herbal mouthwash was statistically non-significant. The lowest PI, GI, and CFU values were obtained in the subjects of group 4.

Conclusion: Herbal mouthwashes are a promising alternative and are effective in maintaining oral hygiene.

## Introduction

Nearly 50% of people worldwide are affected by oral diseases that go untreated. A clear indication that many people lack access to appropriate oral health care, which includes prevention, risk protection, and restorative and rehabilitative services, is the increase of 1 billion cases globally over the past 30 years [1]. Although it may not be clinically apparent during the course of the disease, in most cases, gingivitis precedes periodontitis, contributing further to attachment loss [2].

Dental plaque is a complex biofilm harboring a plethora of microorganisms on the surface of the teeth; therefore, plaque control is critical for sustaining good oral hygiene. Plaque accumulation can be checked using various mechanical and chemical methods, but research indicates inadequate plaque removal using mechanical methods alone [3]. Mechanical plaque control procedures are focused on tooth surfaces, whereas the microbial accumulation on soft tissues remains unaddressed and may contribute to aggravating gingivitis and periodontitis [3]. This contrast with studies that indicate that mechanical removal of bacterial plaque is adequate [3].

Many over-the-counter products are available on the market to aid oral hygiene maintenance, and the gold standard among them is chlorhexidine (CHX) mouthwash [1-5]. Several untoward effects of CHX, like altered taste perception, bitter taste, discoloration of teeth or restorations, and so on, have been reported [4]. However, the possibility of oral bacteria developing resistance to CHX has also been seen [5]. So, the aim of the study was to evaluate the efficacy of alternative herbal mouthwashes.

## Materials and methods

The study was conducted in collaboration with various private clinics and involved 125 healthy volunteers aged 18-25 years. Ethical clearance was obtained from the College of Dental Sciences and Research Centre with the registration of an institutional review board number: CDSRC/2020/321, and informed consent was obtained from all the dentists and the participating volunteers. The volunteers were screened based on rigorous inclusion and exclusion criteria, following which they were subjected to the preparatory protocol of the study.

Inclusion criteria included age (18 to 24 years), good oral health status (mean of all scored surfaces), a dentition consisting of ≥20 teeth with a minimum of 5 teeth in each quadrant, including decayed, missing, and filled teeth (DMFT index value ≥2, plaque index ≤ 2, gingival index ≤1), and the absence of periodontal disease. Exclusion criteria excluded patients with any systemic disease, patients consuming antibiotics in the last two weeks, patients with poor oral hygiene or periodontal disease, patients receiving any dental treatment during the period of the study, a history of hypersensitivity or adverse reactions to any material used in the study, and a history of recent tooth extraction.

Sample size calculation

A sample size of 120 was obtained by using SPSS software based on α error = <5% (p<0.05) and β = 80%. Based on this calculation, the minimum sample size required in each group was found to be 25 subjects. The subjects were selected based on the specified criteria mentioned above.

Preparatory phase

Patients were informed about the study and its protocol. A history was obtained, a clinical examination was performed, and zero baseline values were achieved by performing professional scaling and polishing. The plaque was measured using the Modified Quigley-Hein Plaque Index (PI), and gingival health was assessed using the gingival index (GI) [6,7]. Examination at all stages of the study was performed by a single, trained examiner to prevent bias. Colony-forming unit (CFU) values were obtained to quantify the amount of Streptococcus mutant bacteria in saliva. Stimulated salivary samples were obtained from patients 45 minutes after the use of mouthwash and immersed in thioglycollate broth. Inoculation was done on Mitis-Salivarius agar modified by adding bacitracin and sucrose at concentrations of 0.2 units/ml and 20% w/v, respectively. The agar plates were incubated under anaerobic conditions for four days at 37 °C. Enumeration of colonies was done using a stereomicroscope and colony counting grid (Hi-Media Laboratories, Mumbai, India).

All of the subjects were put into five groups based on the mouthwash that would be used. Group 1: negative control - distilled water; group 2: positive control - hexidine mouthwash (ICPA, Gujarat, India); group 3: FeelFresh herbal mouthcare gargle (Able Exports, Gujarat, India), group 4: HiOra mouthwash (Himalaya Wellness Company, Bengaluru, India); group 5: Colgate MaxFresh fresh tea mouthwash (Colgate-Palmolive, India). All subjects were asked to use 15 mL of the allocated mouthwash for 30 seconds, twice daily, after breakfast and dinner, with an interval of 12 hours in between. They were instructed to continue their routine oral hygiene procedures daily using a toothbrush and dental floss and were asked to refrain from using any additional oral hygiene products or techniques, including chewing gum. After 7 and 30 days, the examination was performed, and PI, GI, and CFU values were obtained for all subjects. On the concluding day, all the subjects received professional tooth polishing [1,3,4]. The obtained data were compiled, tabulated, and analyzed statistically by applying ANOVA and post-hoc Tukey’s tests, respectively, using SPSS software version 22 (IBM Corp., Armonk, NY).

## Results

Age group were in range from 21 to 43 years. An equal number of males and females were included in the study to eliminate gender-related bias. History was obtained, clinical examination was performed and baseline values were brought to zero by performing professional scaling and polishing. The descriptive statistics representing the mean and standard deviation (SD) are indicated in Table 1.

**Table 1:** Mean standard deviation for all the groups PI: plaque index; GI: gingival index; CFU/ML: colony-forming unit per millilitre

Group	7 days			30 days		
Description	PI	GI	CFU/ML	PI	GI	CFU/ML
Negative control	0.483 (0.039)	0.33 (0.039)	182.757 (7.582)	0.535 (0.029)	0.204 (0.039)	122.617 (6.369)
Group 1 - distilled water						
Positive control	0.525 (0.083)	0.181 (0.048)	141.257 (4.599)	0.271 (0.027)	0.007 (0.001)	68.273 (0.054)
Group 2 - hexidine						
Neem	0.470 (0.032)	0.313 (0.040)	139.13 (3.366)	0.274 (0.036)	0.093 (0.026)	58.17 (3.187)
Group 3 - FeelFresh						
S. persica	0.431 (0.049)	0.274 (0.029)	148.36 (4.093)	0.224 (0.019)	0.093 (0.026)	38.06 (4.720)
Group 4 - HiOra						
Colgate Maxfresh	0.418 (0.039)	0.232 (0.049)	149.393 (3.852)	0.244 (0.038)	0.087 (0.028)	73.18 (3.875)
Group 5 - Colgate MaxFresh						

The lowest values of PI at 7 and 30 days are seen in the Colgate MaxFresh group and the Hiora group, respectively. The lowest values of GI are seen in the hexidine group at both 7 and 30 days. The lowest values of CFU/mL at 7 and 30 days are seen in the Feelfresh group and the Hiora group, respectively.

Table 2 demonstrates the comparison among groups, indicating a statistically non-significant difference among groups, including the negative control (p=0.43).

**Table 2:** Intergroup comparison to evaluate statistically significant difference among groups including negative control (p-value < 0.05 - statistically significant difference) PI: plaque index; GI: gingival index; CFU: colony-forming unit

ANOVA followed by Tukey’s post-hoc test	Groups					P-value
	Negative control (group 1)	Positive control (group 2)	Feel Fresh (group 3)	Hiora (group 4)	Colgate MaxFresh (group 5)	0.43
							PI	2.52	7.04	7.652	4.324	1.885
GI	5.53	1.525	1.151	1.265	3.896	
CFU	5.57	5.76	3.425	8.459	6.565	

## Discussion

The primary step in achieving good oral hygiene is the removal of dental plaque. Mechanical plaque removal measures include the use of toothbrushes, dental floss, interdental brushes, etc., but their efficacy is questionable in the inaccessible and proximal areas of the oral cavity. In a study, when compared to the baseline for both toothbrushes, there were statistically significant decreases in mean gingival and plaque ratings at each visit (p<0.05). At each visit, there were no statistically significant variations in the scores between the two toothbrushes (p>0.05) [8].

In terms of lowering GI or plaque in the medium term, moderately and lowly certain evidence indicated no difference between manual and electric toothbrushes (GI: MD 0.02, 95% CI −0.06 to 0.09; plaque: standardized mean difference 0.29, 95% CI −0.07 to 0.65; 2 RCTs, 120 participants). Short-term results were inconsistent (four RCTs; evidence with low to extremely low certainty) [9]. While these measures aid in the removal of dental plaque adhering to the supragingival surfaces, the subgingival areas and soft tissues remain unaddressed, thereby resulting in inflammation of the tissues and periodontal disease [1,3]. Consequentially, chemical plaque removal using mouthwash has been recommended as an adjunctive oral hygiene measure [1,3,4,10].

The use of CHX and essential oils as a mouthwash has been approved by the American Dental Association, and CHX has been the gold standard ever since. CHX exhibits bactericidal action at 0.2% concentration and antiplaque action at 0.12% concentration. The presence of a positively charged bis-biguanide molecule enables adhesion to the negatively charged bacterial and mucosal surfaces, thereby resulting in bactericidal action. It is well-known for its substantivity, which results in an effective and extended release of the agent for up to seven days, consequently decreasing the bacterial counts of cariogenic species like *S. mutans*. However, several untoward effects have been noted with the use of CHX, like staining and altered taste perception, following which the quest for an alternative agent began [4-6,8,9]. In the present study, CHX showed the highest reduction in the values of the gingival index, validating its positive impact on oral health. Contrary to this result, a study done by Abdulkareem et al. found additionally, hyaluronic acid (HA) may be a useful substitute for CHX due to its higher level of participant acceptance. The participants appeared to prefer the HA mouthwash above the other mouthwashes, according to the analysis of participant input on the different mouthwashes [11].

Hiora mouthwash consists of herbal derivatives like *Salvadora persica*, *Terminalia bellerica*, piper betel, and *Elletaria cardamom*, which are known for their anti-cariogenic, anti-inflammatory, immunity-boosting, and antiplaque effects. The active components in *Salvadora perisca* include trimethylamine, salvadorine, fluoride, silica, sulfur, vitamin C, saponins, flavonoids, sterols, etc., which exert a bactericidal effect [8,12,13]. Tannins inhibit the enzyme glucosyltransferase, resulting in decreased plaque adhesion. Additionally, owing to their astringent effect, tannins also help to reduce gingivitis [14]. The chloride component prevents calculus formation and aids in stain removal from the tooth surfaces [14]. The presence of *Terminalia bellirica* promotes tissue healing and repair, while essential oils increase the salivary flow rate, subsequently increasing the buffering action of saliva [12,13]. Furthermore, the presence of ursane and glycosides in peppermint provides an additional astringent and anti-inflammatory effect [8,11-16]. In the present study, HiOra mouthwash showed a reduction in the values of indices, which may be attributed to the synergistic action of its components. While it has shown reduced readings for PI and bacterial count, the difference in comparison with CHX is statistically nonsignificant.

Neem, the chief component of Feelfresh mouthwash, has been identified as *Azadirachta indica* in the modern era and has been known to bear plentiful active molecules possessing therapeutic benefits since ancient times [17,18]. The primary compound azadirachtin exerts an antibacterial effect by interfering with oxidative phosphorylation in bacterial mitochondria, resulting in respiratory chain inhibition [19,20]. Additional antibacterial effects are seen due to the presence of chemical compounds like alkaloids and flavonoids, which inhibit nucleic acid synthesis and energy metabolism by inhibiting the enzyme dihydrofolate reductase [20]. Glycosides and steroid compounds alter the permeability of the cell membrane, resulting in leakage of cellular contents and cell lysis, furthering the bactericidal action [20]. Nimbin, Nimbidin, Nimbidol, and Nimbolinin, along with salannin and quercetin, cause damage to the cell membrane, thereby inhibiting bacterial growth [20]. In the present study, the lowest bacterial count was observed in the Feelfresh group at both seven-day intervals, with a statistically non-significant difference in comparison to other groups, including negative controls. The above-mentioned properties of neem corroborate these findings, suggesting its beneficial effect on oral health. Although neem-based mouthwashes have a bitter taste and may result in reduced patient compliance, it is noteworthy that their benefits far outweigh their disadvantages.

Green tea (GT) is a non-fermented tea drink that is known for its health benefits around the world [21,22]. It is considerably more palatable than CHX and *A. indica* [21-23]. The active component is a polyphenol, namely epigallo-catechin-3-gallate (EGCG), known to display antimicrobial, anti-inflammatory, anti-bacterial, and anti-carcinogenic activity [24]. GT is known to act against Gram-positive and Gram-negative bacteria [25]. On local application, it reduces the acidity of plaque and saliva in addition to reducing the salivary levels of virulent cariogenic pathogens like *S. mutans* and lactobacilli. It promotes the secretion of protective constituents like immunoglobulins, lactoferrin, lysosomes, histatin, and mucin in saliva. By inhibiting enzymes like glucosyltransferase and salivary amylase, GT reduces plaque adhesion and fermentable carbohydrates, thereby reducing plaque index scores [21-23]. It suppresses the acidogenicity and aciduricity of *S. mutans*, thereby reducing the negative impact of cariogenic bacteria [26-29]. All of these effects highlight the greater anticarcinogenic potential of GT, which might explain the results in the present study, where GT, while demonstrating the lowest values of PI at seven days, was equally efficient as other groups, including negative controls.

In the present study, the negative control group representing distilled water also demonstrated a reduction in PI, GI, and bacterial count values despite the absence of any active antibacterial or antimicrobial agent. Furthermore, the Hawthorne effect, which suggests that participation in a clinical trial results in improved health regardless of the treatment received, may have contributed to the improved oral health observed in the subjects of the negative control group [8].

The study's limitations are the small sample size, and this kind of research is essential if we are to completely understand the advantages mouthwashes can have in terms of health problems. The more researchers who work on this, the more obvious the solutions. For assessing the effectiveness and safety of mouthwash, additional larger clinical studies are required.

## Conclusions

Herbal mouthwashes are a promising alternative that is effective in maintaining oral hygiene, especially in light of the drawbacks that have been observed in connection with CHX. The findings of this research led the authors to the conclusion that using mouthwash as an adjunct to procedures that involve mechanically removing plaque is beneficial.

## References

[1] Pai MR, Acharya LD, Udupa N (2022). Global oral health status report: towards universal health coverage for oral health by 2030. J Ethnopharmacol.

[2] Kim J, Amar S (2006). Periodontal disease and systemic conditions: a bidirectional relationship. Odontology.

[3] Soben P (2006). Essentials of preventive and community dentistry. BMC Complement Altern Med.

[4] Nayak N, Varghese J, Shetty S (2019). Evaluation of a mouthrinse containing guava leaf extract as part of comprehensive oral care regimen: a randomized placebo-controlled clinical trial. BMC Complement Altern Med.

[5] Cieplik F, Jakubovics NS, Buchalla W, Maisch T, Hellwig E, Al-Ahmad A (2019). Resistance toward chlorhexidine in oral bacteria - is there cause for concern?. Front Microbiol.

[6] Turesky S, Gilmore ND, Glickman I (1970). Reduced plaque formation by the chloromethyl analogue of victamine C. J Periodontol.

[7] Loe H, Silness J (1963). Periodontal disease in pregnancy. I. Prevalence and severity. Acta Odontol Scand.

[8] Mamat N, Mani SA, Danaee M (2022). T-shaped toothbrush for plaque removal and gingival health in children: a randomized controlled trial. BMC Oral Health.

[9] Ferozali F, Johnson G, Cavagnaro A (2007). Health benefits and reductions in bacteria from enhanced oral care. Spec Care Dentist.

[10] Safiaghdam H, Oveissi V, Bahramsoltani R, Farzaei MH, Rahimi R (2018). Medicinal plants for gingivitis: a review of clinical trials. Iran J Basic Med Sci.

[11] Abdulkareem AA, Al Marah ZA, Abdulbaqi HR, Alshaeli AJ, Milward MR (2020). A randomized double-blind clinical trial to evaluate the efficacy of chlorhexidine, antioxidant, and hyaluronic acid mouthwashes in the management of biofilm-induced gingivitis. Int J Dent Hyg.

[12] Sayal D, Sood R, Yadav N, Jha B, Dodwad V (2016). Comparing the effect of three different mouthwashes cranberry, HIORA and chlorhexidine on the management of periodontal diseases. Int J Periodontol Implantol.

[13] Ramamurthy JA, Visha MG (2018). Comparison of effect of hiora mouthwash versus chlorhexidine mouthwash in gingivitis patients: a clinical trial. Asian J Pharm Clin Res.

[14] Wali BN, Afif IK, Balata JF, Badr NA (2018). Effect of an experimental mouth wash extracted from Salvadora persica on cariogenic bacteria and dental plaque formation. Int J Curr Res.

[15] Garapati B, Ramamurthy J, Shanmugam R (2022). Formulation, development, and evaluation of anti-inflammatory and antimicrobial effects of a novel polyherbal mouthwash-An in vitro study. J Popul Ther Clin Pharmacol.

[16] Amir Alireza RG, Afsaneh R, Seied Hosein MS (2014). Inhibitory activity of Salvadora persica extracts against oral bacterial strains associated with periodontitis: An in-vitro study. J Oral Biol Craniofac Res.

[17] Botelho MA, Santos RA, Martins JG (2008). Efficacy of a mouthrinse based on leaves of the neem tree (Azadirachta indica) in the treatment of patients with chronic gingivitis: a double-blind, randomized, controlled trial. J Med Plants Res.

[18] Tidke S, Chhabra GK, Madhu PP, Reche A, Wazurkar S, Singi SR (2022). The effectiveness of Herbal versus Non-Herbal mouthwash for periodontal health: a literature review. Cureus.

[19] Nimbulkar G, Garacha V, Shetty V, Bhor K, Srivastava KC, Shrivastava D, Sghaireen MG (2020). Microbiological and clinical evaluation of neem gel and chlorhexidine gel on dental plaque and gingivitis in 20-30 years old adults: a randomized parallel-armed, double-blinded controlled trial. J Pharm Bioallied Sci.

[20] Abdurrahman M, Lestari ES, Prihatiningsih T (2022). The effectiveness of ethanol extract of neem leaf (Azadirachta indica) mouthwash against the growth of Streptococcus sp. J Biomed Transl Res.

[21] Botten D, Fugallo G, Fraternali F, Molteni C (2015). Structural properties of green tea catechins. J Phys Chem B.

[22] Gupta DA, Bhaskar DJ, Gupta RK (2014). Green tea: a review on its natural antioxidant therapy and cariostatic benefts. Biol Sci Pharm Res.

[23] Jigisha A, Nishant R, Navin K, Pankaj G (2012). Green tea: a magical herb with miraculous outcomes. Int Res J Pharm.

[24] Cho YS, Schiller NL, Kahng HY, Oh KH (2007). Cellular responses and proteomic analysis of Escherichia coli exposed to green tea polyphenols. Curr Microbiol.

[25] Reygaert WC (2014). The antimicrobial possibilities of green tea. Front Microbiol.

[26] Vilela MM, Salvador SL, Teixeira IG, Del Arco MC, De Rossi A (2020). Efficacy of green tea and its extract, epigallocatechin-3-gallate, in the reduction of cariogenic microbiota in children: a randomized clinical trial. Arch Oral Biol.

[27] Romoozi E, Bekhradi R, Talebi M, Barouti P, Kamali E (2018). Effect of green tea mouthwash on reducing plaque and gingivitis. J Dent Health Oral Disord Ther.

[28] Nagar A, Tyagi A, Gupta SJ, Banga A, Sharma N, Gupta A (2018). Comparative evaluation of the anti-plaque efficacy of green tea extract mouthrinse and white tea extract mouthrinse with chlorhexidine gluconate mouthrinse-A clinical and microbiological study. J Dent Spec.

[29] Ahmadi MH, Sarrami L, Yegdaneh A, Homayoni A, Bakhtiyari Z, Danaeifar N, Akhlaghi N (2019). Comparative evaluation of efficacy of green tea mouth rinse and green tea gel on the salivary Streptococcus mutans and Lactobacillus Colony count in 12-18-year-old teenagers: a randomized clinical trial. Contemp Clin Dent.

